# Potential of *Aedes albopictus* as a bridge vector for enzootic pathogens at the urban-forest interface in Brazil

**DOI:** 10.1038/s41426-018-0194-y

**Published:** 2018-11-28

**Authors:** Taissa Pereira dos Santos, David Roiz, Filipe Vieira Santos de Abreu, Sergio Luiz Bessa Luz, Marcelo Santalucia, Davy Jiolle, Maycon Sebastiao Alberto Santos Neves, Frédéric Simard, Ricardo Lourenço-de-Oliveira, Christophe Paupy

**Affiliations:** 1 MIVEGEC Laboratory, IRD-CNRS-Montpellier Univ., Montpellier, 34394 France; 2 Oswaldo Cruz Institute, FIOCRUZ, Rio de Janeiro, RJ 21040-900 Brazil; 3 Leonidas and Maria Deane Institute, FIOCRUZ, Manaus, AM 69057-070 Brazil; 4 State of Goias Public Health Laboratory Dr. Giovanni Cysneiros, Goiania, GO 74853-120 Brazil

## Abstract

The invasive species *Aedes albopictus* is present in 60% of Brazilian municipalities, including at the interfaces between urban settings and forests that are zoonotic arbovirus hotspots. We investigated *Ae. albopictus* colonization, adult dispersal and host feeding patterns in the anthropic-natural interface of three forested sites covering three biomes in Brazil in 2016. To evaluate whether an ecological overlap exists between *Ae. albopictus* and sylvatic yellow fever virus (YFV) in forests, we performed similar investigations in seven additional urban-forest interfaces where YFV circulated in 2017. We found *Ae. albopictus* in all forested sites. We detected eggs and adults up to 300 and 500 m into the forest, respectively, demonstrating that *Ae. albopictus* forest colonization and dispersal decrease with distance from the forest edge. Analysis of the host identity in blood-engorged females indicated that they fed mainly on humans and domestic mammals, suggesting rare contact with wildlife at the forest edge. Our results show that *Ae. albopictus* frequency declines as it penetrates into the forest and highlight its potential role as a bridge vector of zoonotic diseases at the edge of the Brazilian forests studied.

## Introduction

The risk of the emergence of zoonotic infectious diseases is particularly high in regions under the influence of tropical forests because these important biodiversity hotspots are undergoing anthropogenic land use changes^1^. Landscape anthropization, especially urbanization, shifts the risk of mosquito-borne pathogen emergence by affecting the mosquito and host communities both quantitatively and qualitatively^2,3^. Landscape disturbances at the urban-forest interface may facilitate the dispersion of anthropophilic mosquito species into previously unfavorable habitats^4^, thus modifying vector-host interactions and potentially leading to more contact with sylvatic (i.e., enzootic) reservoirs of zoonotic pathogens. In this scenario, opportunistic mosquito species can act as bridge vectors between sylvatic and urban pathogen transmission cycles.

During the last 35 years, *Aedes* (*Stegomyia*) *albopictus* (Skuse) (Diptera: Culicidae) has expanded geographically from Southeast Asia to other continents, withimportant human health consequences and risks related particularly to arbovirus transmission^5–9^. This invasive mosquito species, which was considered sylvatic in its native area, has adapted to human settings (domestication) by exploiting man-made water containers as larval habitats and humans and/or domestic animals as its main blood source^10^. Domestication is one of the key features (together with other biological traits, such as desiccation-resistant eggs and overwintering ability) that facilitated the successful expansion of *Ae. albopictus* throughout anthropogenic environments worldwide^7^. Nevertheless, it remains unclear to what extent invasive (and “domesticated”) *Ae. albopictus* populations have preserved their capacity to colonize forest environments. Indeed, *Ae. albopictus* was previously detected at the forest border of rural or urban habitats in colonized areas^5,11,12^, and its opportunistic blood feeding behavior has been extensively described (i.e., a wide range of vertebrate hosts with a marked preference for mammals, especially humans, compared to birds)^13–15^. In opportunistic species, the degree of blood feeding on domestic or wild vertebrates relative to humans is strongly dependent on the local host availability^16^. Importantly, *Ae. albopictus* can support the transmission of epidemic arboviruses (e.g., chikungunya, dengue, and Zika viruses) and possibly many other enzootic and zoonotic arboviruses^5,17–19^. In addition, experimental vector competence studies and viral genome detection or isolation in nature indicated that *Ae. albopictus* can transmit several enzootic arboviruses (e.g., La Crosse Virus, West Nile Virus, Eastern equine encephalomyelitis, Cache Valley Virus, Keystone Virus, Potosi Virus, Tensaw Virus, Chandipura, Jamestown Canyon, Orungo, Rift Valley, Ross River, Oropuche virus, Mayaro virus, and yellow fever virus (YFV)^20–35^). Its vector competence for several pathogens, its opportunistic feeding behavior, and its capacity to colonize urban, rural and natural habitats suggest that *Ae. albopictus* could be a bridge vector to allow pathogen transfers from animal to human compartments and vice versa^13,36^.

Since its first detection in Brazil in the 1980s, *Ae. albopictus* has geographically expanded to approximately 60% of Brazilian municipalities^37^, across urban, peri-urban and rural environments^38–40^, and in some cases at the border of the Atlantic and Amazon forests^12,41–43^. The Brazilian Amazon forest harbors 187 different arboviruses, among which, 34 are of medical interest^44,45^. Of particular interest is sylvatic YFV, which causes cyclic epidemic waves with high lethality in Brazil. Following its spatial expansion to the Southeast region in late 2016, the YFV territory now largely overlaps with areas of high *Aedes* (*Stegomyia*) *aegypti* (Linnaeus) and *Ae. albopictus* infestation, and this increases the risk of a reemergence of urban transmission throughout South America^37,46–48^. However, few studies have focused on *Ae. albopictus* colonization and dispersion in Brazil. Some studies inside forests were carried out as part of the entomological surveillance during yellow fever epizootics, or as part of ecological projects on native mosquitoes^42,49^. However, systematic investigations are needed to measure *Ae. albopictus* dispersal penetration into neotropical forests to understand its potential as a bridge vector in relation to the emergence risk of zoonotic arboviruses in bordering Brazilian cities and elsewhere. A higher risk occurs when mammalian diversity is elevated, as it is the case for the Atlantic and Cerrado forests, which are considered hotspots for biodiversity conservation^50^.

Here we studied the capacity of *Ae. albopictus* to colonize and disperse into forested environments from the edge (i.e., modified environment) to deeper locations (i.e., more preserved environment) in three Brazilian biomes, the Amazon, Cerrado and Atlantic forests. We also analyzed mosquito blood meals to determine *Ae. albopictus* host feeding patterns and potential interactions with wild vertebrates.

## Results

### *Ae. albopictus* forest colonization

**Fig. 1:**
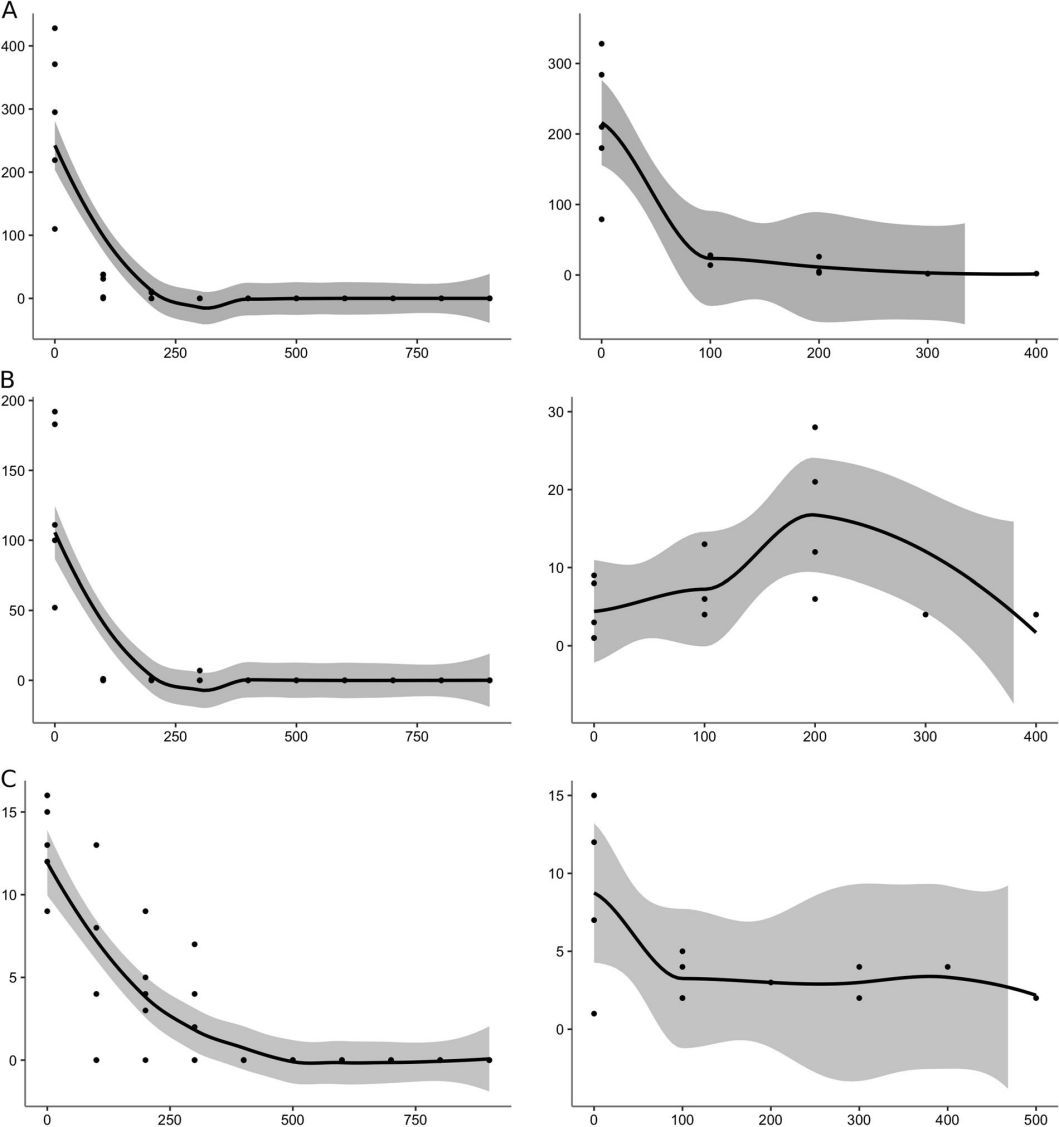
Charts in ggplot with LOESS smoothing showing the total number of eggs (left panels) and *Ae. albopictus* females (right panels) on the y-axis as a function of the distance (in meters) from the forest edge (x-axis) at the (**a**) Adolpho Ducke, (**b**) Pedra Branca, and (**c**) Morro dos Macacos sampling sites. The shaded area represents the 95% confidence interval of the mean value (solid line). Statistical details are provided in Table S4

During the 2016 survey, the maximal distance of *Ae. albopictus* egg detection was 300 m from the forest edge, even when sampling was carried out up to 1000 m inside the forest. Specifically, in Adolpho Ducke-Amazonas, the total egg number per distance (five ovitraps per distance) rapidly decreased from 0 to 200 m from the edge (0 m: 1423, 100 m: 118, 200 m: 20 eggs) (Fig. 1). Similarly, at Morro dos Macacos-Goias and Pedra Branca-Rio de Janeiro, eggs were found up to 300 m (0 m: 65, 100 m: 25, 200 m: 21, 300 m: 13 at Morro dos Macacos, and 0 m: 638, 100 m: 2, 200 m: 1, 300 m: 7 at Pedra Branca). Overall, the number of eggs decreased significantly (*p* < 0.00001) with distance from the forest edge in all sampled areas (explained deviance of more than 70%) (Tables S2 and S4).

In the areas surveyed in 2017, mosquito eggs were detected up to the maximal sampling distance (300 m from the forest edge) at four sites: Belo Horizonte-Minas Gerais (0 m: 9, 100 m: 99), Simonésia-Minas Gerais (0 m: 5, 100 m: 55, 200 m: 23, 300 m: 109), Domingos Martins-Espirito Santo (0 m: 26, 100 m: 42, 200 m: 2, 300 m: 17), and Salvador-Bahia (0 m: 236, 100 m: 117, 200 m: 4, 300 m: 64). Although technical problems prevented egg counting in Casimiro de Abreu-Rio de Janeiro and Maricá-Rio de Janeiro, positive ovitraps were detected at all distances (Table S2). Eggs from Serra- Espirito Santo were destroyed during transport.

In Adolpho Ducke and Salvador, all hatched eggs belonged to *Ae. albopictus*. In Pedra Branca, 1.6% of all hatched eggs collected at 0 m corresponded to *Ae. aegypti*, and in Belo Horizonte, 10% of the eggs collected at 100 meters were from *Haemagogus leucocelaenus*. All hatched eggs collected in Maricá, Casimiro de Abreu and those collected at 200 m in Simonésia and at 300 m in Domingos Martins belonged to sylvatic mosquito species (Table S2).

### *Ae. albopictus* forest dispersal

In Adolpho Ducke, BG-Sentinel traps collected 1,232 *Ae. albopictus* adults (768 females and 464 males) with a mean number of specimens per trap and per day (MN/T/D) of 7 (SD = 8.35). At Morro dos Macacos and Pedra Branca, 63 (59 females and 4 males) and 139 (129 females and 10 males) adult *Ae. albopictus* specimens were collected, respectively (MN/T/D: 1.43 ± 0.81 and 2.07 ± 1.68, respectively). At these three sites, *Ae. albopictus* adults were sampled from the edge to 500 meters into the forest. At Morro dos Macacos, 49% of all *Ae. albopictus* specimens were trapped at 0 meter, with two females trapped at 500 m. At Adolpho Ducke, 89% of females were collected at 0 meter, with two females collected at 400 m. At Pedra Branca, 48% of females were sampled at 200 m, with seven specimens collected at 400 m.

Along of the Adolpho Ducke transect, the abundance of *Ae. albopictus* females was significantly (*p* < 0.00001) associated with distance, decreasing from 0 meters up to 400 m, with an explained deviance of 30%. Conversely, in Pedra Branca, *Ae. albopictus* abundance increased up to 200 m, and then decreased up to 400 m. Nevertheless, the distance explained the presence of adults in the forest (*p* < 0.00001; explained deviance of 55.20%). In Morro dos Macacos, the number of *Ae. albopictus* significantly (*p* < 0.00001) decreased with distance and the distance explained the presence of adults in the forest (explained deviance of 48.32%) (Fig. 1 and online Tables S3 and S4 for statistical details).

In 2017, adult *Ae. albopictus* specimens were trapped as far as 300 m (limit of trap deployment) at all sites, with the exception of Casimiro de Abreu and Maricá in the Atlantic forest. The total number of adults caught across the 300 m transects varied from two females in Casimiro de Abreu to 43 females in Salvador. Similarly, only females were trapped in Belo Horizonte (*n* = 12), Domingos Martins (*n* = 5), Maricá (*n* = 7) and Serra (*n* = 3). At Simonésia, 28 specimens were collected (20 females and 8 males) (Table S3).

### *Ae. albopictus* blood meal analysis

**Fig. 2:**
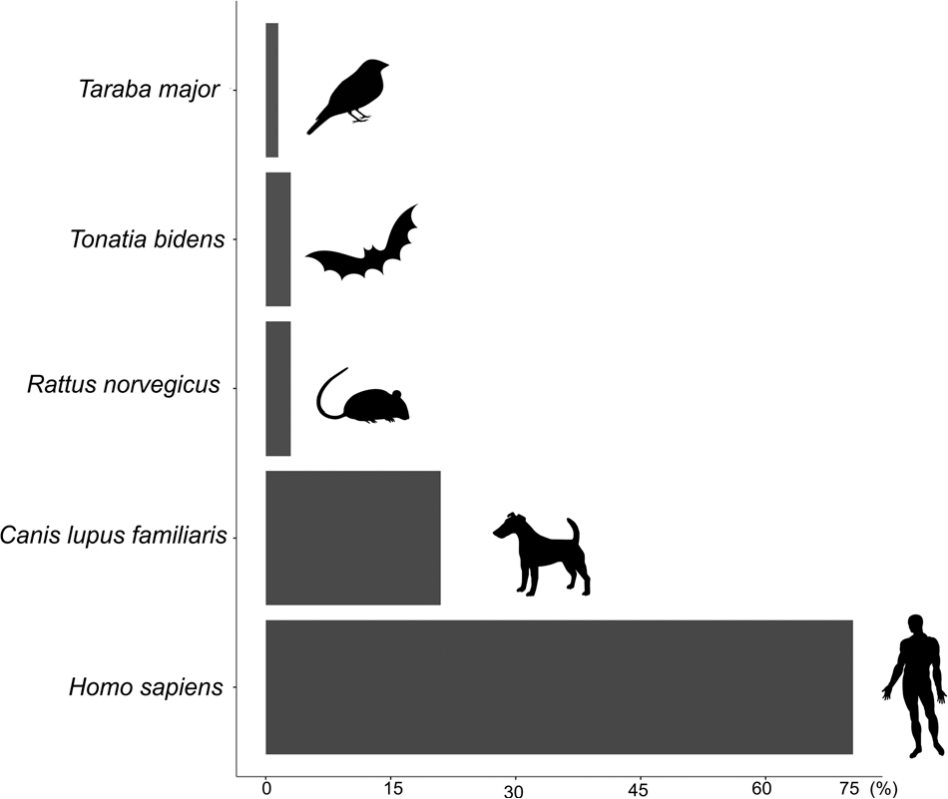
Horizontal bar chart in ggplot showing the number of vertebrate species found in the blood meals of *Ae. albopictus* specimens. The bar graph value was calculated using data from 66 *Ae. albopictus* specimens analyzed without distinction of area or collection distance from the forest edge

Among all the trapped *Ae. albopictus* females, 66 were engorged with blood (*n* = 61 in Adolpho Ducke, *n* = 1 in Morro dos Macacos, and *n* = 4 in Pedra Branca). Most of them (91%) were collected at the forest edge (online Table S5) and very few were collected from deeper in the forest (five at 100 m in Adolpho Ducke; one at 200 m in Pedra Branca). Molecular analyses indicated that most blood meals (98%) were taken from mammals, mainly humans (71%), dogs (21%), brown rats (*Rattus norvegicus*, 3%) and greater round-eared bats (*Tonatia bidens*, 3%) (Fig. 2, online Table S5). Only one mosquito was found to have fed on a bird (great antshrike, *Taraba major*, 1.5%). No engorged *Ae. albopictus* were captured in the seven additional sampling sites.

## Discussion

Here, we studied the establishment of *Ae. albopictus* at the edge of and inside Brazilian forests as well as its feeding habits to understand the potential risk of this invading mosquito transferring zoonotic arboviruses to humans. Its abundance, opportunistic trophic behavior and vector competence for several viruses contributes to its potential role in the spill-over of zoonotic pathogens from sylvatic hosts to humans, or to domestic animals and then potentially to humans^13,15^.

We observed that forest colonization by *Ae. albopictus* rapidly decreased with distance from the edge (200–300 m inside the forest). Together with previous findings, these result confirm that *Ae. albopictus* population density declines as they penetrate into the forest^10,51^. Although *Ae. albopictus* was captured at all surveyed forests both before and during the YFV outbreak, its dispersal seems to be somewhat limited because adults were not sampled beyond 500 m from the forest edge. The number of trapped adults usually decreased with the distance from the edge of the forest, with the exception of Pedra Branca, where the abundance of *Ae. albopictus* females increased up to 200 m and then sharply decreased up to 400 m, probably due to the presence of human visitors between 0–200 m at this reserve (personal observation). The presence of humans in these areas could represent a blood source for feeding, as well as a source of breeding/oviposition sites (i.e., plastic containers left behind). *Aedes albopictus* colonization and dispersal occurs deeper into Brazilian secondary forests, as described previously in a one-year study at Represa do Cigano in Rio de Janeiro where the eggs of wild and rubidium chloride-marked females were detected up to 1000 m inside the anthropized area of the Tijuca Forest^12,41^.

The blood meal identifications showed a clear pattern of mammalian host preference (especially humans), as reported in several previous field studies^13–15,52^. In addition to humans, blood meals were found to be from domestic or commensal hosts (dogs and brown rats) and, less frequently, from wild hosts (two bats and one bird), confirming the opportunistic feeding behavior of *Ae. albopictus*^5,13,14^. The greater round-eared bat is an omnivorous species that is usually found in hollow trees at low heights and hunts in clearings (modified environments and forest edges). The great antshrike is a bird that lives in clearings and low density vegetation at the forest edge^53,54^. These results highlight that *Ae. albopictus* feeds mostly on humans and domestic animals but also on wildlife at the edge of the forest and suggests host-seeking by flying at low heights. Additional studies should investigate the potential host feeding patterns of *Ae. albopictus* on wildlife living inside forest patches.

Our study and previous data^12,41^ suggest that, in Brazil, *Ae. albopictus* can occupy the few hundred meters beyond the forest edge, but then its density progressively decreases with distance from the edge. This could be explained by a reduction in the availability of its key resources, which are common at the forest edge, such as suitable breeding sites and host abundance. Therefore, the degree of forest fragmentation and anthropization could be a relevant factor that modulates *Ae. albopictus* colonization and dispersal. Alternatively, *Ae. albopictus* populations could be maladapted to sylvatic environments (i.e., the species has lost its ancestral biological trait). The presence of competitors, predators or parasites in the natural larval habitats in neotropical forests could also explain this observation.

Although the forest edge could be considered an ‘emergence area’ where zoonotic diseases can spill-over from zoonotic reservoirs to humans via *Ae. albopictus*, the present results suggest that the risk of transfer from wildlife is less likely than from domestic commensal hosts. This must be confirmed by additional studies, particularly on the interactions between *Ae. albopictus* and monkeys that are YFV reservoirs. Natural YFV infections in the *Ae. albopictus* specimens captured in the Cerrado biome of Brazil during the 2016–2018 YFV outbreak were recently confirmed^48^, suggesting that such interactions occurred. Moreover, it was previously shown that some Brazilian *Ae. albopictus* populations, including those living at the sites surveyed here (Manaus, Goiania and Rio de Janeiro), are competent to support the transmission of several YFV strains^26,55^. Furthermore, in vivo experiments have demonstrated that YFV can rapidly adapt to this vector species^56^. In this study, *Ae. albopictus* females were trapped up to 300 m in five of the seven surveyed forests where active enzootic YFV transmission was reported in 2017, as well as at the forest edge of all the surveyed forests. Our data suggests an ecological overlap between *Ae. albopictus* and YFV reservoirs. It is crucial that future studies better evaluate the propensity of this mosquito to bite monkeys that live in the canopy, thus helping the transfer of sylvatic YFV to humans and the emergence of a rural/intermediate cycle in Brazil, or the reemergence of the urban cycle.

Taken together, these results highlight the potential role of *Ae. albopictus* as a bridge vector for zoonotic diseases in the human-animal interface at the edge of Brazilian forests. Its potential participation in the spill-over of dozens of zoonotic arboviruses harbored in Brazilian forests from sylvatic or ubiquitous hosts directly to humans, from wildlife to commensal and domestic hosts, and between humans in the ecotone and natural and modified environments, as well as rural areas, deserves to be investigated. For instance, capuchins, marmosets, opossums and other commensal and ubiquitous mammals that live and find shelter in low vegetation and/or on the ground at the forest edge are amplifiers of arboviruses with great epidemic potential (e.g., YFV, Mayaro virus), and their territory overlaps with that of *Ae. albopictus* in Brazil. Understanding the mechanisms of disease emergence will allow for the development of early detection and control programs to reduce disease incidences and economic burdens.

## Materials and methods

### Study areas

**Fig. 3:**
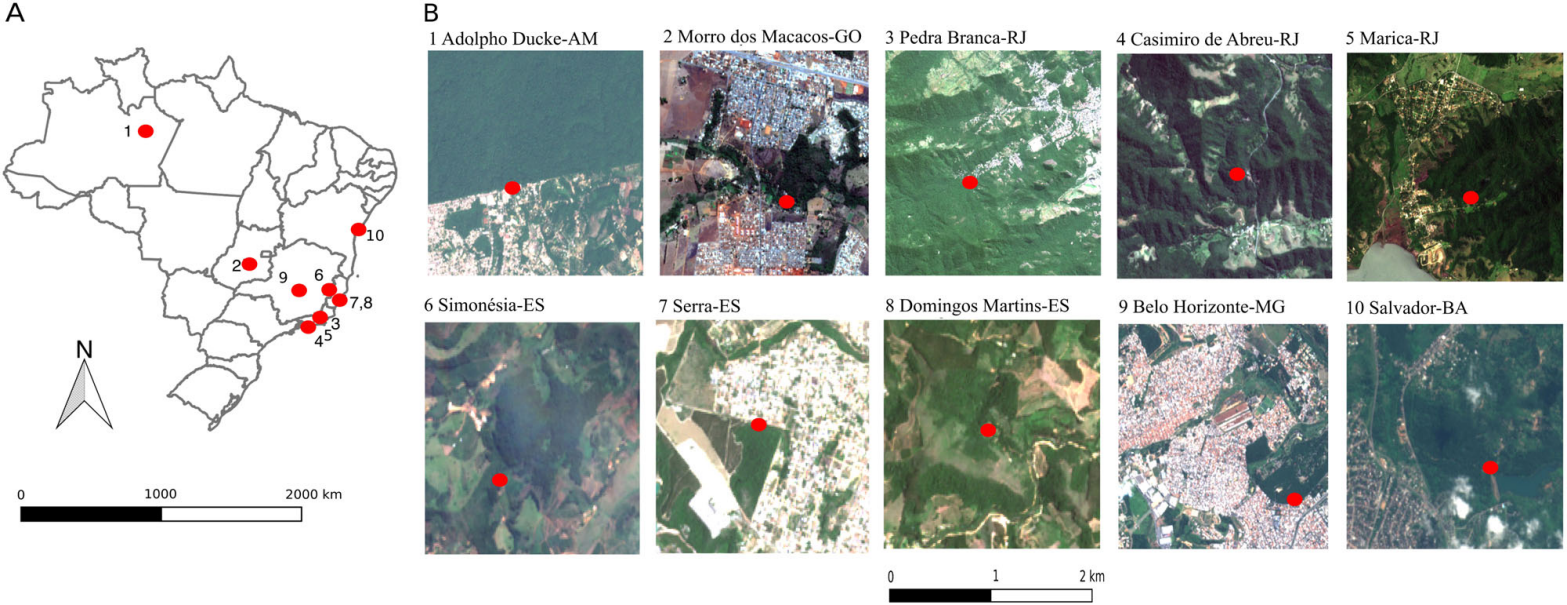
*Ae. albopictus* sampling sites at ten urban-forest interfaces in Brazil. **a** Localization of the ten sites in Brazil; **b** Satellite images showing each sampling site as a red dot. Satellite images were downloaded from https://earthexplorer.usgs.gov and the maps were drawn using Qgis 2.18.14

Surveys were undertaken before (from January to May 2016) and during the 2017–2018 severe YFV outbreak (from February to June 2017). In 2016, mosquitoes were sampled in three urban-forest interfaces located in the cities of Manaus (Adolpho Ducke site, 3°00′12.78″S; 59°55’37.86”W, January 7 to March 3), Goiania (Morro dos Macacos site, 6°40′16.32″S; 49°22′49.93″W, April 17 to May 27), and Rio de Janeiro (Pedra Branca site, 22°56′6.57″S; 43°26′42.19″W, from March 4 to April 16) (Fig. 3 and online Table S1). In 2017, we extended our survey by including seven supplementary urban-forest interfaces located in four Brazilian states where YFV was circulating. In addition to increasing the number of study sites to have a broader and more generalizable view of the *Ae. albopictus* colonization in Brazilian forests, the aim was also to assess the potential ecological overlap between *Ae. albopictus* and sylvatic YFV. The additional sites were Belo Horizonte (19°51′59.29″; 44°0′43.51, from February 10 to 13), Simonésia (19°55′12.06″; 41°54′20.23″”, from February 16 to 18) in the Cerrado biome, Domingos Martins (20°17′12.48″; 40°50′14.35″, from February 21 to 23), Serra (20°6′46.89″; 40°11′12.53″, from March 14 to 17), Casimiro de Abreu (22°26′33.31″; 42°12'30.34″, from March 28 to 31), Maricá (22°55′24.44″; 42°42′27.88″, from May 4 to 8) and Salvador (12°49′50.34″; 38°27′18.77″, from June 6 to 8) in the Atlantic forest (Fig. 3).

### Study design

The field entomological survey was undertaken according to an anthropization gradient from the edge to the interior of the forest. At the three long-term surveyed sites (Adolpho Ducke, Morro dos Macacos, and Pedra Branca), the mosquito sampling design was based on a grid of five parallel lines spaced 25 m apart and intersected perpendicularly by a grid of ten parallel lines spaced 100 m apart, extending from the edge (0 meters) to 900 m inside the forest (online Figure S1). Field work was organized into two sampling phases with two different methods to differentiate between *Ae. albopictus* colonization (first sampling phase) and dispersion (second sampling phase). The term “colonization” refers to the capacity to exploit forest environments for suitable oviposition sites. The term “dispersion” was used to measure the abundance of host-seeking female mosquitoes in the forest. The first sampling phase (10 consecutive days) was conducted by deploying 50 ovitraps (one at each intersection between parallel and perpendicular lines) made up of a dark plastic cup with 300 ml of hay infusion and one wooden paddle for mosquito egg collection^57^. Every 5 days, paddles were replaced and the recovered paddles were transported to the laboratory for egg counting on day 10. In the laboratory, paddles were immersed in water for egg hatching, larval breeding and identification of emerging adults using appropriate morphological taxonomic keys^58^. The second sampling phase (7 consecutive days) was carried out using 15 BG-Sentinel traps (Biogents) baited with a combination of BG-lure and dry ice as source of CO_2_ to capture host-seeking adult mosquitoes at different distances up to 500 meters inside the forest. The number of BG-Sentinel traps per distance varied due to operational limitations. Traps operated beginning at 8:00am on day 1 until 5:00pm on day 7 and were monitored every 24 h by replacing the collection bags containing mosquitoes and dry ice. We defined a “sampling cycle” as the 10 days of ovitrap monitoring followed by the 7 days of BG-Sentinel trap monitoring. Two sampling cycles were carried out in Adolpho Ducke and Morro dos Macacos and one was completed in Pedra Branca. The sampling method and approach used in the seven forest interfaces surveyed during the 2017 YFV outbreak were essentially the same, except that 12 BG-Sentinel traps were operated from 0 to 300 m from the edge of the forest for 5 days, considering the preliminary results of the 2016 sampling. Ovitraps were installed at the same points as the BG-Sentinel traps for 10 days.

### Trophic behavior analysis

Blood-engorged *Ae. albopictus* females collected with the BG-Sentinel traps were used to identify the blood-feeding patterns in forest environments. DNA was extracted from abdomens containing blood and used for PCR amplification according to the protocol by Bitome-Essono et al.^59^. Specific primers were used to amplify a portion of the 16 S rRNA gene or to amplify a portion of the cytochrome B oxidase gene, if the first PCR test failed. Genomic DNA from a mosquito engorged with rabbit blood was used as positive control. DNase-free water was used as negative control. All PCR-amplified products (10 μl) were run on 2% agarose gels in TBE buffer and positive samples were sent to GENEWIZ for forward and reverse sequencing after purification. Consensus sequences were compared with existing sequences using the nucleotide BLAST database to determine the host species, according to Bitome-Essono et al.^59^.

### Statistical analysis

Data from the Adolpho Ducke, Pedra Branca and Morro dos Macacos forests were used to study (1) the relationship between the presence of eggs and the distance from the edge (colonization), and (2) the relationship between the presence of *Ae. albopictus* females and the distance from the edge (dispersion). After completing the first exploratory data analysis, a negative binomial generalized linear model was developed. An offset variable was included to weigh for the adult mosquito sampling effort. Statistical analyses were performed using the R studio 3.3.1 tool in the R software, version 1.0.143. Data from the other seven study sites could not be analyzed due to the low number of sampled mosquitoes.

## Electronic supplementary material

Supplementary information
