# Supplementary material for: Potential of Aedes albopictus as a bridge vector for enzootic pathogens at the urban-forest interface in Brazil
Source: Emerg Microbes Infect. 2018 Nov 28;7:191. doi: 10.1038/s41426-018-0194-y (PMC6258732; doi:10.1038/s41426-018-0194-y)
Supplement: Supplementary file 1 — Supplementary information [file 41426_2018_194_MOESM1_ESM.docx]

**Supplementary information**

**Potential of *Aedes albopictus* as a bridge vector for enzootic pathogens at the urban-forest interface in Brazil**

Taissa Pereira dos Santos^1*^, David Roiz^1*^, Filipe Vieira Santos de Abreu^2^, Sergio Luiz Bessa Luz^3^, Marcelo Santalucia^4^, Davy Jiolle^1^, Maycon Sebastiao Alberto Santos Neves^2^, Frédéric Simard^1^, Ricardo Lourenço-de-Oliveira^2^, Christophe Paupy^1^

**Author affiliations**: ^1^MIVEGEC Laboratory, IRD-CNRS-Montpellier Univ., Montpellier, 34394, France; ^2^Oswaldo Cruz Institute, FIOCRUZ, Rio de Janeiro, RJ, 21040-900, Brazil; ^3^Leonidas and Maria Deane Institute, FIOCRUZ, Manaus, AM, 69057-070 Brazil; ^4^State of Goias Public Health Laboratory Dr. Giovanni Cysneiros, Goiania, GO, 74853-120, Brazil.

^*^These authors contributed equally to this article.

**Correspondence:** Christophe Paupy; Taissa Pereira dos Santos

**E-mails**: [christophe.paupy@ird.fr](mailto:christophe.paupy@ird.fr); [tayssadnz@gmail.com](mailto:tayssadnz@gmail.com).

**Table S1:** Geographic coordinates of the seven additional collection sites.

| **Area** | **Latitude** | **Longitude** |
| --- | --- | --- |
| Belo Horizonte | 19°51'59.29" | 44° 0'43.51 |
| Simonésia | 19°55'12.06" | 41°54'20.23" |
| Domingos Martins | 20°17'12.48" | 40°50'14.35" |
| Serra | 20° 6'46.89" | 40°11'12.53" |
| Casimiro de Abreu | 22°26'33.31" | 42°12'30.34" |
| Salvador | 12°49'50.34" | 38°27'18.77" |
| Maricá | 22°55'24.44" | 42°42'27.88" |

**Table S2:** Summary of egg sampling yields with ovitraps in the different sampling areas and according to the distance from the forest edge.

| **2016 samplings** | | | | |
| --- | --- | --- | --- | --- |
| **Area** | **Distance** | **Egg number** | **Hatching** | **Other species** |
| Adolpho Ducke | 0 | 1423 | 102 | 0 |
|  | 100 | 108 | 24 | 0 |
|  | 200 | 20 | 0 | 0 |
|  | 300 to 900 | 0 | 0 | 0 |
| Subtotal | - | 1551 | 126 | 0 |
| Pedra Branca | 0 | 638 | 183 | 3^b^ |
|  | 100 | 2 | 0 | 0 |
|  | 200 | 1 | 0 | 0 |
|  | 300 | 7 | 0 | 0 |
|  | 400 to 900 | 0 | 0 | 0 |
| Subtotal | - | 648 | 183 | 3 |
| Morro dos Macacos | 0 | 65 | 14 | 0 |
|  | 100 | 25 | 0 | 0 |
|  | 200 | 21 | 0 | 0 |
|  | 300 | 13 | 0 | 0 |
|  | 400 to 900 | 0 | 0 | 0 |
| Subtotal | - | 124 | 14 | 0 |
| **2017 samplings** | | | | |
| Domingos Martins | 0 | 26 | 0 | 0 |
|  | 100 | 42 | 0 | 0 |
|  | 200 | 2 | 0 | 0 |
|  | 300 | 17 | 0 | 13 ^c^ |
| Subtotal | - | 87 | 0 | 13 ^c^ |
| Salvador | 0 | 236 | 29 | 0 |
|  | 100 | 117 | 12 | 0 |
|  | 200 | 4 | 0 | 0 |
|  | 300 | 64 | 0 | 0 |
| Subtotal | - | 421 | 41 | 0 |
| Simonésia | 0 | 5 | 0 | 0 |
|  | 100 | 55 | 2 | 0 |
|  | 200 | 23 | 0 | 2 ^c^ |
|  | 300 | 109 | 11 | 0 |
| Subtotal |  | 192 | 13 | 2 ^c^ |
| Belo Horizonte | 0 | 9 | 8 | 0 |
|  | 100 | 99 | 18 | 2^c^ |
|  | 200 | 0 | 0 | 0 |
|  | 300 | 0 | 0 | 0 |
| Subtotal | - | 108 | 26 | 2 |
| Maricá ^a^ | 0 | ND | 0 | 173^c^ |
|  | 100 | ND | 0 | 36^c^ |
|  | 200 | ND | 0 | 212^c^ |
|  | 300 | ND | 0 | 19^c,d^ |
| Subtotal |  | _ | 0 | 440 ^c,d^ |
| Casimiro de Abreu^a^ | 0 | ND | 2 | 8^c^ |
|  | 100 | ND | 0 | 1^e^ |
|  | 200 | ND | 0 | 4^d^ |
|  | 300 | ND | 0 | 1^d^ |
| Subtotal |  |  | 2 | 14^c,d,e^ |

Hatching: number of *Aedes albopictus* emerged from collected eggs. Other species: number of other mosquito species emerged from the collected eggs. a: Technical problems prevented egg counting in Casimiro de Abreu and Maricá; b: *Ae. aegypti;* c: *Hg. leucocelaenus* d: *Ae. terrens;* e: *Hg. janthinomys.* ND: Not done.**Table S3:** Summary of adult mosquito sampling with BG-Sentinel traps in the different areas and according to the distance from the forest edge.

| **2016 Sampling** | | | | | | | | | |
| --- | --- | --- | --- | --- | --- | --- | --- | --- | --- |
| **Area** | **Distance** | **TN** | **DF** | **TNF** | **DFF** | **FF** | **TNM** | **DFM** | **FM** |
| Adolpho Ducke | 0 | 1096 | 0.89 | 638 | 0.55 | 0.62 | 431 | 0.34 | 0.38 |
|  | 100 | 98 | 0.08 | 64 | 0.05 | 0.65 | 34 | 0.03 | 0.35 |
|  | 200 | 32 | 0.025 | 16 | 0.01 | 0.5 | 16 | 0.01 | 0.5 |
|  | 300 | 4 | 0.003 | 3 | 0 | 0.75 | 1 | 0 | 0.25 |
|  | 400 | 2 | 0.002 | 2 | 0 | 1 | 0 | 0 | 0 |
|  | 500 | 0 | 0 | 0 | 0 | 0 | 0 | 0 | 0 |
| Subtotal | - | 1232 | 1 | 768 | 0.62 | 0.62 | 464 | 0.38 | 0.37 |
| Pedra Branca | 0 | 27 | 0.19 | 26 | 0.19 | 0.96 | 1 | 0.01 | 0.04 |
|  | 100 | 29 | 0.2 | 27 | 0.19 | 0.93 | 2 | 0.01 | 0.07 |
|  | 200 | 67 | 0.48 | 61 | 0.44 | 0.91 | 6 | 0.04 | 0.09 |
|  | 300 | 9 | 0.06 | 9 | 0.06 | 1 | 0 | 0 | 0 |
|  | 400 | 7 | 0.05 | 6 | 0.04 | 0.85 | 1 | 0.01 | 0.15 |
|  | 500 | 0 | 0 | 0 | 0 | 0 | 0 | 0 | 0 |
| Subtotal | - | 139 | 1 | 129 | 0.93 | 0.96 | 10 | 0.07 | 0.04 |
| Morro dos Macacos | 0 | 31 | 0.49 | 28 | 0.44 | 0.90 | 3 | 0.05 | 0.09 |
|  | 100 | 17 | 0.26 | 17 | 0.27 | 1 | 0 | 0 | 0.09 |
|  | 200 | 3 | 0.04 | 3 | 0.05 | 1 | 0 | 0 | 0 |
|  | 300 | 6 | 0.09 | 5 | 0.08 | 0.83 | 1 | 0.02 | 0.17 |
|  | 400 | 4 | 0.06 | 4 | 0.06 | 1 | 0 | 0 | 0 |
|  | 500 | 2 | 0.03 | 2 | 0.03 | 1 | 0 | 0 | 0 |
| Subtotal | - | 63 | 1 | 59 | 0.94 | 0.93 | 4 | 0.06 | 0.07 |
| **2017 Sampling** | | | | | | | | | |
| Domingos Martins | 0 | 2 | 0.40 | 2 | 0.40 | 1 | 0 | 0 | 0 |
|  | 100 | 1 | 0.20 | 1 | 0.20 | 1 | 0 | 0 | 0 |
|  | 200 | 0 | 0 | 0 | 0 | 0 | 0 | 0 | 0 |
|  | 300 | 2 | 0.40 | 2 | 0.40 | 1 | 0 | 0 | 0 |
| Subtotal | - | 5 | 1 | 5 | 1 | 1 | 0 | 0 | 0 |
| Salvador | 0 | 14 | 0.33 | 14 | 0.33 | 1 | 0 | 0 | 0 |
|  | 100 | 11 | 0.26 | 11 | 0.26 | 1 | 0 | 0 | 0 |
|  | 200 | 6 | 0.14 | 6 | 0.14 | 1 | 0 | 0 | 0 |
|  | 300 | 12 | 0.28 | 12 | 0.28 | 1 | 0 | 0 | 0 |
| Subtotal | - | 43 | 1 | 43 | 1 | 1 | 0 | 0 | 0 |
| Simonésia | 0 | 0 | 0 | 0 | 0 | 0 | 0 | 0 | 0 |
|  | 100 | 6 | 0.21 | 4 | 0.14 | 0.67 | 2 | 0.07 | 0.33 |
|  | 200 | 6 | 0.21 | 6 | 0.21 | 1 | 0 | 0 | 0 |
|  | 300 | 16 | 0.57 | 10 | 0.36 | 0.63 | 6 | 0.21 | 0.37 |
| Subtotal | - | 28 | 1 | 20 | 0.71 | 0.71 | 8 | 0.29 | 0.29 |
| Belo Horizonte | 0 | 2 | 0.16 | 2 | 0.16 | 1 | 0 | 0 | 0 |
|  | 100 | 3 | 0.25 | 3 | 0.25 | 1 | 0 | 0 | 0 |
|  | 200 | 0 | 0 | 0 | 0 | 0 | 0 | 0 | 0 |
|  | 300 | 7 | 0.58 | 7 | 0.58 | 1 | 0 | 0 | 0 |
| Subtotal | _ | 12 | 1 | 12 | 1 | 1 | 0 | 0 | 0 |
| Serra | 0 | 1 | 0.33 | 1 | 0.33 | 1 | 0 | 0 | 0 |
|  | 100 | 1 | 0.33 | 1 | 0.33 | 1 | 0 | 0 | 0 |
|  | 200 | 0 | 0 | 0 | 0 | 0 | 0 | 0 | 0 |
|  | 300 | 1 | 0.33 | 1 | 0.33 | 1 | 0 | 0 | 0 |
| Subtotal | - | 3 | 1 | 3 | 1 | 3 | 0 | 0 | 0 |
| Maricá | 0 | 0 | 0 | 0 | 0 | 0 | 0 | 0 | 0 |
|  | 100 | 3 | 0.43 | 3 | 0.43 | 1 | 0 | 0 | 0 |
|  | 200 | 4 | 0.57 | 4 | 0.57 | 1 | 0 | 0 | 0 |
|  | 300 | 0 | 0 | 0 | 0 | 0 | 0 | 0 | 0 |
| Subtotal | - | 7 | 1 | 7 | 1 | 1 | 0 | 0 | 0 |
| Casimiro de Abreu | 0 | 2 | 1 | 2 | 1 | 1 | 0 | 0 | 0 |
|  | 100 | 0 | 0 | 0 | 0 | 0 | 0 | 0 | 0 |
|  | 200 | 0 | 0 | 0 | 0 | 0 | 0 | 0 | 0 |
|  | 300 | 0 | 0 | 0 | 0 | 0 | 0 | 0 | 0 |
| Subtotal | - | 2 | 1 | 2 | 1 | 1 | 0 | 0 | 0 |

TN: Total number of captured adult *Ae. albopictus* specimens. DF: Number of *Ae. albopictus* by distance divided by the total number of mosquitoes collected in that area. TNF: Total number of captured females. DFF: Number of *Ae. albopictus* by distance divided by the total number of mosquitoes collected in that area. FF: Frequency of *Ae. albopictus* females per distance. TNM: Total number of captured *Ae. albopictus* males. DFM: Number of *Ae. albopictus* males by distance divided by the total number of mosquitoes collected in that area. FM: Frequency of *Ae. albopictus* males per distance.

**Table S4:** Results of the generalized additive model with negative binomial distribution (GLM.NB) for the Adolpho Ducke, Pedra Branca, and Morro dos Macacos sampling data according to the distance from the forest edge.

| Zone | Dep | Ind. | Estimate | SE | z-value | Pr (>\|z\|) | Dev.Explained | Dispersion |
| --- | --- | --- | --- | --- | --- | --- | --- | --- |
| Egg abundance (GLM.NB) | | | | | | | | |
| AD | Ab | Intercept | 5.71 | 0.473 | 12.048 | < 2e-16 *** | 91.82% | 0.33 |
|  |  | Distance | -0.02 | 0.003 | -7.69 | 2.03e-14 *** |  |  |
| PB | Ab | Intercept | 4.28 | 0.779 | 5.45 | 3.87e-08 *** | 78.92% | 0.68 |
|  |  | Distance | -0.01 | 0.003 | -4.610 | 4.03e-06 *** |  |  |
| MM | Ab | Intercept | 2.88 | 0.337 | 8.54 | <2e-16 *** | 74.60% | 0.66 |
|  |  | Distance | -0.01 | 0.001 | -6.62 | 3.41e-11 *** |  |  |
| Adult *Aedes albopictus* abundance (GLM.NB) | | | | | | | | |
| AD | Ab | Intercept | 2.294 | 0.099 | 23.070 | < 2e-16 *** | 30% | 1.401 |
|  |  | Distance | -0.008 | 0.001 | -5.943 | 2.79e-09 *** |  |  |
|  | Ab+offset | Intercept | -2.693 | 0.098 | -27.435 | <2e-16 *** | 3% | 1.436 |
|  |  | Distance | 0.0009 | 0.001 | 0.726 | 0.468 |  |  |
| PB | Ab | Intercept | 0.607 | 0.162 | 3.728 | 0.0001 *** | 1.3% | 1.10 |
|  |  | Distance | 0.001 | 0.0008 | 1.514 | 0.129 |  |  |
|  | Ab+offset | Intercept | -4.581 | 0.190 | -24.050 | < 2e-16 *** | 55.29% | 1.47 |
|  |  | Distance | 0.009 | 0.001 | 9.223 | < 2e-16 *** |  |  |
| HM | Ab | Intercept | 0.415 | 0.164 | 2.530 | 0.001 * | 6.9% | 0.51 |
|  |  | Distance | -0.006 | 0.0008 | -0.712 | 0.476 |  |  |
|  | Ab+offset | Intercept | -4.13 | 0.212 | -19.460 | < 2e-16 *** | 48.32% | 1.27 |
|  |  | Distance | 0.006 | 0.001 | 5.399 | 6.69e-08 *** |  |  |

AD: Adolpho Ducke; PB: Pedra Branca; MM: Morro dos Macacos; Dep: dependent variable; Ind: independent variable, Dev: deviance; Ab: abundance; SE, standard error.

**Table S5:** Summary of the molecular analyses to assess the host feeding behavior of blood-engorged *Ae. albopictus* females.

| Area | Dist | Ab | Amplified region | Vertebrate | MS | TS | QC | E value | Id | Accession Nb |
| --- | --- | --- | --- | --- | --- | --- | --- | --- | --- | --- |
| AD | 0 | 1 | 16s | *C. l. familiaris* | 137 | 137 | 100% | 3.00E-29 | 98% | MK128940 |
|  |  | 1 | 16s | *C. l. familiaris* | 171 | 171 | 96% | 3.00E-39 | 98% | MK128941 |
|  |  | 1 | 16s | *C. l. familiaris* | 167 | 167 | 94% | 5.00E-38 | 98% | MK128942 |
|  |  | 1 | 16s | *C. l. familiaris* | 172 | 172 | 97% | 1.00E-39 | 98% | MK128943 |
|  |  | 1 | 16s | *C. l. familiaris* | 143 | 143 | 100% | 6.00E-31 | 99% | MK128944 |
|  |  | 1 | 16s | *C. l. familiaris* | 113 | 113 | 96% | 6.00E-22 | 87% | MK128945 |
|  |  | 1 | 16s | *C. l. familiaris* | 169 | 169 | 95% | 1.00E-38 | 98% | MK128946 |
|  |  | 1 | 16s | *C. l. familiaris* | 165 | 165 | 96% | 2.00E-37 | 97% | MK128947 |
|  |  | 1 | 16s | *C. l. familiaris* | 167 | 167 | 96% | 5.00E-38 | 98% | MK128948 |
|  |  | 1 | 16s | *C. l. familiaris* | 134 | 134 | 92% | 5.00E-28 | 92% | MK128949 |
|  |  | 1 | 16s | *C. l. familiaris* | 145 | 145 | 97% | 2.00E-31 | 98% | MK128950 |
|  |  | 1 | 16s | *C. l. familiaris* | 172 | 172 | 95% | 1.00E-39 | 99% | MK128951 |
|  |  | 1 | 16s | *C. l. familiaris* | 165 | 165 | 96% | 2.00E-37 | 97% | MK128952 |
|  |  | 1 | 16s | *C. l. familiaris* | 147 | 147 | 100% | 4.00E-32 | 100% | MK128953 |
|  |  | 1 | 16s | *Homo sapiens* | 171 | 171 | 100% | 3.00E-39 | 96% | MK128901 |
|  |  | 1 | 16s | *Homo sapiens* | 174 | 174 | 100% | 3.00E-40 | 97% | MK128902 |
|  |  | 1 | 16s | *Homo sapiens* | 172 | 172 | 100% | 9.00E-40 | 97% | MK128903 |
|  |  | 1 | 16s | *Homo sapiens* | 176 | 176 | 97% | 8.00E-41 | 98% | MK128905 |
|  |  | 1 | 16s | *Homo sapiens* | 167 | 167 | 93% | 5.00E-38 | 97% | MK128906 |
|  |  | 1 | 16s | *Homo sapiens* | 161 | 161 | 93% | 2.00E-36 | 97% | MK128908 |
|  |  | 1 | 16s | *Homo sapiens* | 182 | 182 | 99% | 2.00E-42 | 98% | MK128910 |
|  |  | 1 | 16s | *Homo sapiens* | 178 | 178 | 95% | 2.00E-41 | 99% | MK128911 |
|  |  | 1 | 16s | *Homo sapiens* | 141 | 141 | 100% | 2.00E-30 | 95% | MK128912 |
|  |  | 1 | 16s | *Homo sapiens* | 119 | 119 | 98% | 1.00E-23 | 88% | MK128913 |
|  |  | 1 | 16s | *Homo sapiens* | 178 | 178 | 94% | 2.00E-41 | 99% | MK128914 |
|  |  | 1 | 16s | *Homo sapiens* | 178 | 178 | 93% | 2.00E-41 | 99% | MK128915 |
|  |  | 1 | 16s | *Homo sapiens* | 132 | 132 | 100% | 1.00E-27 | 94% | MK128916 |
|  |  | 1 | 16s | *Homo sapiens* | 182 | 182 | 98% | 2.00E-42 | 98% | MK128917 |
|  |  | 1 | 16s | *Homo sapiens* | 180 | 180 | 98% | 6.00E-42 | 98% | MK128918 |
|  |  | 1 | 16s | *Homo sapiens* | 134 | 134 | 96% | 5.00E-28 | 91% | MK128919 |
|  |  | 1 | 16s | *Homo sapiens* | 172 | 172 | 97% | 1.00E-39 | 97% | MK128920 |
|  |  | 1 | 16s | *Homo sapiens* | 178 | 178 | 95% | 2.00E-41 | 99% | MK128921 |
|  |  | 1 | 16s | *Homo sapiens* | 182 | 182 | 97% | 2.00E-42 | 99% | MK128922 |
|  |  | 1 | 16s | *Homo sapiens* | 134 | 134 | 98% | 4.00E-28 | 95% | MK128923 |
|  |  | 1 | 16s | *Homo sapiens* | 172 | 172 | 94% | 1.00E-39 | 98% | MK128924 |
|  |  | 1 | 16s | *Homo sapiens* | 170 | 170 | 93% | 4.00E-39 | 98% | MK128925 |
|  |  | 1 | 16s | *Homo sapiens* | 172 | 172 | 92% | 1.00E-39 | 98% | MK128926 |
|  |  | 1 | 16s | *Homo sapiens* | 152 | 152 | 94% | 1.00E-33 | 95% | MK128927 |
|  |  | 1 | 16s | *Homo sapiens* | 168 | 168 | 99% | 2.00E-38 | 96% | MK128928 |
|  |  | 1 | 16s | *Homo sapiens* | 172 | 172 | 93% | 1.00E-39 | 98% | MK128929 |
|  |  | 1 | 16s | *Homo sapiens* | 170 | 170 | 93% | 4.00E-39 | 98% | MK128930 |
|  |  | 1 | 16s | *Homo sapiens* | 140 | 140 | 92% | 7.00E-30 | 94% | MK128931 |
|  |  | 1 | 16s | *Homo sapiens* | 172 | 172 | 94% | 1.00E-39 | 98% | MK128932 |
|  |  | 1 | 16s | *Homo sapiens* | 165 | 165 | 98% | 2.00E-37 | 97% | MK128933 |
|  |  | 1 | 16s | *Homo sapiens* | 134 | 134 | 100% | 2.00E-28 | 95% | MK128935 |
|  |  | 1 | 16s | *Homo sapiens* | 170 | 170 | 93% | 4.00E-39 | 98% | MK128936 |
|  |  | 1 | 16s | *Homo sapiens* | 156 | 156 | 94% | 1.00E-34 | 97% | MK128937 |
|  |  | 1 | 16s | *Homo sapiens* | 63.9 | 63.9 | 43% | 5.00E-07 | 97% | MK128938 |
|  |  | 1 | CYTB | *Homo sapiens* | 569 | 569 | 97% | 1.00E-158 | 99% | MK153182 |
|  |  | 1 | CYTB | *Homo sapiens* | 573 | 573 | 96% | 1.00E-159 | 99% | MK153183 |
|  |  | 1 | CYTB | *Homo sapiens* | 586 | 586 | 99% | 1.00E-163 | 99% | MK153185 |
|  |  | 1 | CYTB | *Homo sapiens* | 582 | 582 | 96% | 2.00E-162 | 100% | MK153187 |
|  |  | 1 | CYTB | *Homo sapiens* | 579 | 579 | 98% | 2.00E-161 | 99% | MK153189 |
|  |  | 1 | 16s | *Rattus norvegicus* | 106 | 106 | 95% | 1.00E-19 | 87% | MK128956 |
|  |  | 1 | CYTB | *Rattus norvegicus* | 398 | 398 | 98% | 6.00E-107 | 90% | MK153191 |
|  |  | 1 | 16s | *Tonatia bidens* | 82.4 | 82.4 | 60% | 1.00E-12 | 96% | MK128954 |
|  |  | 1 | CYTB | *Taraba major* | 466 | 466 | 90% | 2.00E-127 | 96% | MK153190 |
|  |  | 1 | 16s | *Tonatia bidens* | 78.8 | 78.8 | 62% | 1.00E-11 | 96% | MK128955 |
|  | 100 | 1 | 16s | *Homo sapiens* | 150 | 150 | 100% | 4.00E-33 | 93% | MK128909 |
|  |  | 1 | 16s | *Homo sapiens* | 87.8 | 87.8 | 88% | 4.00E-14 | 82% | MK128934 |
|  |  | 1 | CYTB | *Homo sapiens* | 577 | 577 | 99% | 8.00E-161 | 98% | MK153188 |
| MM | 0 | 1 | 16s | *Homo sapiens* | 167 | 167 | 93% | 5.00E-38 | 97% | MK128939 |
| PB | 0 | 1 | 16s | *Homo sapiens* | 161 | 161 | 99% | 2.00E-36 | 94% | MK128907 |
|  | 100 | 1 | CYTB | *Homo sapiens* | 592 | 592 | 97% | 3.00E-165 | 100% | MK153184 |
|  |  | 1 | CYTB | *Homo sapiens* | 564 | 564 | 96% | 6.00E-157 | 99% | MK153186 |
|  | 200 | 1 | 16s | *Homo sapiens* | 169 | 169 | 94% | 1.00E-38 | 98% | MK128904 |

Area: sampling area; Dist: distance from the forest edge; Ab: Number of abdomens from *Ae. albopictus* females analyzed separately; MS: Maximum score; TS: total score; QC: query cover; E value and Id (identity): values obtained with the BLAST search (NCBI website); Acession Nb: Genebank accession numbers; AD: Adolpho Ducke; MM: Morro dos Macacos; PB: Pedra Branca; 16s, 16S rRNA gene; CYTB, cytochrome B gene.

**Figure S1:** Mosquito sampling design implemented at the three long-term surveyed sites (Adolpho Ducke, Morro dos Macacos, and Pedra Branca)


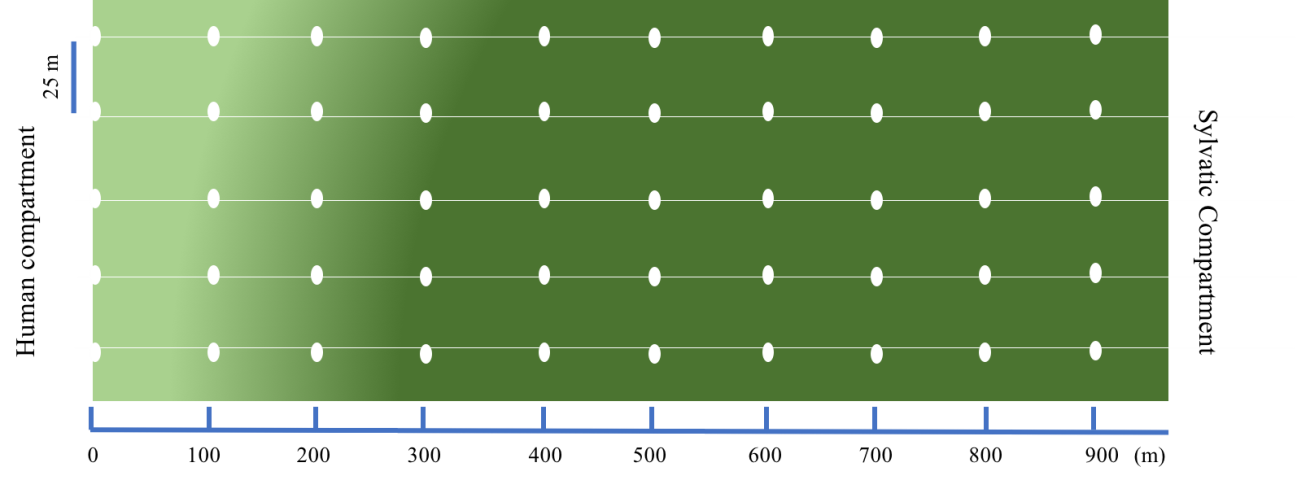
The five horizontal lines intersect ten vertical distances from the forest edge into the forest to investigate the limits of *Ae. albopictus* penetration. The white dots represent the collection sites approximately every 100 meters.
